# UV oxidation of cyclic AMP receptor protein, a global bacterial gene regulator, decreases DNA binding and cleaves DNA at specific sites

**DOI:** 10.1038/s41598-020-59855-x

**Published:** 2020-02-20

**Authors:** Fabian Leinisch, Michele Mariotti, Sofie Hagel Andersen, Søren Lindemose, Per Hägglund, Niels Erik Møllegaard, Michael J. Davies

**Affiliations:** 1 Department of Biomedical Sciences, Panum Institute, University of Copenhagen, Copenhagen, 2200 Denmark; 2 Department of Cellular and Molecular Medicine, Panum Institute, University of Copenhagen, Copenhagen, 2200 Denmark

**Keywords:** Chemical biology, Biophysical chemistry, Post-translational modifications, Proteomics, DNA, Proteomics

## Abstract

UV light is a widely-employed, and environmentally-sensitive bactericide but its mechanism of action is not fully defined. Proteins are major chromophores and targets for damage due to their abundance, but the role of proteins in inducing damage to bound DNA, and the effects on DNA-protein interactions is less well characterized. In *E. coli* (and other Gram-negative bacteria) the cyclic AMP receptor protein (CRP/CAP) regulates more than 500 genes. In this study we show that exposure of isolated dimeric CRP-cAMP to UV modifies specific Met, Trp, Tyr, and Pro side-chains, induces inter-protein Tyr63-Tyr41 cross-links, and decreases DNA binding via oxidation of Met114/Pro110 residues in close proximity at the CRP dimer interface. UV exposure also modifies DNA-bound cAMP-CRP, with this resulting in DNA cleavage at specific G/C residues within the sequence bound to CRP, but not at other G/C sites. Oxidation also increases CRP dissociation from DNA. The modifications at the CRP dimer interface, and the site-specific DNA strand cleavage are proposed to occur via oxidation of two species Met residues (Met114 and Met189, respectively) to reactive persulfoxides that damage neighbouring amino acids and DNA bases. These data suggest that modification to CRP, and bound DNA, contributes to UV sensitivity.

## Introduction

The cyclic AMP receptor protein (CRP) (also known as the catabolite activator protein, CAP) is a global regulator of gene expression in *E. coli* and other Gram negative bacteria. CRP regulates more than 500 genes in *E. coli* by binding to approximately 300 high-affinity sites^1^. CRP also binds to more than 10000 low-affinity sites in the *E. coli* genome, indicating that CRP is not only a specific transcriptional regulator, but also a chromosome shaping protein^2,3^. CRP binds as a dimer to DNA sites, and subsequently with RNA polymerase to activate transcription. This activity is cAMP dependent, with complexation resulting in a conformational transition that converts apoCRP from a low- to a high-affinity DNA binding protein which binds at specific sequences upstream of the promoter^4^. The structural basis of CRP interactions with cAMP is well understood^5^. CRP is a homo-dimeric protein with each subunit able to bind one cAMP in a large N-terminal domain that is also responsible for subunit-subunit interactions^6,7^. A smaller C-terminal domain contains a helix-turn-helix motif involved in DNA attachment, with binding generating a strong kink in the DNA chain^8^. This CRP-DNA binding domain is highly conserved across many bacterial regulatory proteins^9,10^.

Cell killing can also be readily induced by high energy radiation, UV and visible light in the presence of a sensitizer^11,12^. Proteins are both major UV absorbing species^13–15^, and major targets for oxidation due to their high abundance^16^. Trp, Tyr, Met, Cys, cystine and His side-chains are particularly prone to modification by UV light, or oxidants (e.g. singlet oxygen, ^1^O_2_) generated by energy transfer from other excited states^13,14,16^.

We have therefore examined the hypothesis that absorption of UV wavelengths (UVB and some UVA) by the cAMP/CRP complex results in: a) specific side-chain alterations and protein cross-links, b) CRP modifications that modulate protein-DNA complex formation, and c) that illumination of pre-formed cAMP-CRP-DNA complexes results in protein-driven DNA damage and dissociation of bound cAMP-CRP. We present data consistent with this hypothesis. These observations support the notion that UV absorption by DNA-associated proteins can generate DNA damage and potentially modulate bacterial gene expression, though this has not been examined in this study.

## Results

### Effect of UV light on the DNA-binding activity of CRP

**Figure 1:**
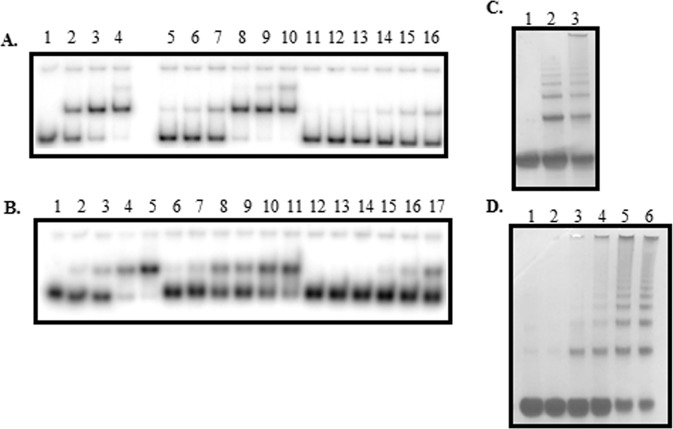
Effects of UV irradiation on CRP-DNA complexes and isolated CRP assessed by gel mobility shift assays (**A**,**B**) and SDS-PAGE (**C**,**D**). Panel A: Gel mobility shift assay of CRP-cAMP complexes (50 µM cAMP) incubated in the dark, or exposed to UV (20 min exposure, λ_max_ 310 nm, 30 nm band width, 6 W m^−2^, normoxia), before addition to ^32^P-labeled DNA in CRP binding buffer (see Materials and methods). DTT (1 mM), NaN_3_ (100 mM) and mannitol (100 mM) were present as indicated. Lane 1: no CRP; lanes 2–4: CRP at 2.5, 5 and 10 nM respectively with no UV exposure; lane 5–7: as lanes 2–4, respectively, but with UV-exposed CRP; lanes 8–10: as lanes 5–7, respectively, but with DTT; lanes 11–13: as lanes 5–7, respectively, but with NaN_3_; lanes 14–16: as lanes 5–7, respectively, but with mannitol. Panel B. Pre-formed CRP-cAMP (50 µM)-DNA complexes were incubated in the dark, or exposed to UV, in the absence or presence of additives (as panel A). Lane 1: no CRP; lanes 2–5: CRP at 1.25, 2.5, 5 and 10 nM, respectively, with no UV exposure; lane 6–8: UV-exposed CRP complex at 2.5, 5 and 10 nM, respectively; lanes 9–11: as lanes 6–8, but with DTT; lanes 12–14: as lanes 6–8, but with NaN_3_; lanes 15–17: as lanes 6–8, but with mannitol. Panel C. SDS-PAGE of isolated CRP (5 μM)-cAMP (50 µM) complex incubated in the dark (lane 1), or exposed to UV (as panel A) for 5 (lane 2) or 20 min (lane 3). Samples were run under reducing conditions, before visualization with InstantBlue staining. Reactions were carried out in buffer containing: 10 mM Tris–HCl (pH 8.0), 50 mM KCl, 2.5 mM MgCl_2_, 1 mM EDTA, 55 µg mL^−1^ bovine serum albumin, 0.05% NP-40 and 2 µg mL^−1^ calf thymus DNA. Panel D. SDS-PAGE analysis of CRP (5 μM)-cAMP (50 µM) complex incubated in the dark, or exposed to UV (as panel A), in the absence or presence of plasmid DNA. Lane 1: CRP alone, no UV; lane 2: CRP with 1 μg plasmid DNA, no UV; lane 3: CRP alone with UV; lanes 4–6: UV-exposed CRP with 0.2, 1 and 3 μg plasmid DNA, respectively. Reactions were carried out in buffer containing:10 mM Tris–HCl (pH 8.0), 50 mM KCl, 2.5 mM MgCl_2_, 1 mM EDTA, 55 µg mL^−1^ bovine serum albumin and 0.05% NP-40.

Gel electrophoretic mobility shift assays showed that biologically-relevant UV wavelengths (280–360 nm, i.e. UVB and some UVA) and doses (6 W m^−2^) alter CRP interactions with a 268 basepair DNA fragment containing a strong symmetric CRP DNA binding site^17^. In the presence of cAMP, and increasing amounts of CRP, a band with lower migration capacity was detected arising from protein binding to the strong symmetric site (Fig. 1A; lanes 2–4). Exposure of the cAMP-CRP complex to UV light, before addition to DNA, markedly decreased protein binding to the DNA (Fig. 1A, compare lanes 2–4 with 5–7), consistent with UV damage impairing protein binding capacity. Inclusion of the thiol compound dithiothreitol (DTT; Fig. 1A, compare lanes 5–7 with 8–10) during UV irradiation prevented the inhibition of CRP binding to DNA. In contrast, NaN_3_ and mannitol did not prevent the inhibition of CRP binding by UV (Fig. 1A, compare lanes 5–7 with 11–13 and 14–16, respectively).

Furthermore, when the pre-assembled cAMP/CRP/DNA complex was subjected to UV irradiation, a similar marked effect of UV was detected using gel shift assays (Fig. 1B, compare lanes 2–5 with 6–8). When present during the UV irradiation of the cAMP/CRP/DNA complex, DTT prevented the loss of CRP binding to DNA; Fig. 1B, compare lanes 6–8 with 9–11). In contrast NaN_3_ (Fig. 1B, lanes 12–14) and mannitol (Fig. 1B, lanes 15–17) provided no protection. These data are consistent with the UV-induced formation of transient reactive species that generate chemical or structural changes to the cAMP-CRP complex that prevent DNA binding.

### Effect of UV light on isolated CRP

The effects of UV exposure on cAMP-CRP composition and structure were examined in both the absence and presence of DNA. Exposure of isolated CRP to increasing UV doses (longer illumination times) resulted in dimer and oligomer formation, as detected by SDS-PAGE. The extent of multimerization, and loss of parent protein, increased with greater UV exposure (Fig. 1C). The absence of O_2_, compared to normoxia, decreased UV-induced CRP oligomerization and loss of the parent protein band (Supplementary Fig. 1A), consistent with a role for O_2_-derived intermediates. Experiments carried out in 80% D_2_O enhanced the loss of the CRP band and increased oligomer formation (Supplementary Fig. 1B), consistent with a role for ^1^O_2_. NaN_3_ decreased oligomer formation particularly when present at high concentration. DTT also decreased oligomer formation but to a lesser extent (Supplementary Fig. 1C). The presence of the poly-histidine tag on the recombinant protein did not impact on oligomer formation (Supplementary Fig. 1D).

Inclusion of plasmid DNA in the cAMP-CRP solutions had no effect on CRP in the absence of UV (Fig. 1D, lanes 1 versus 2), but with UV exposure, the presence of DNA enhanced protein oligomerization, and decreased the intensity of the parent CRP band (Fig. 1D). These data indicate that cAMP-CRP is highly susceptible to UV-mediated damage, and that DNA enhances this damage.

### CRP-bound to DNA gives rise to site specific DNA damage when exposed to UV light

**Figure 2:**
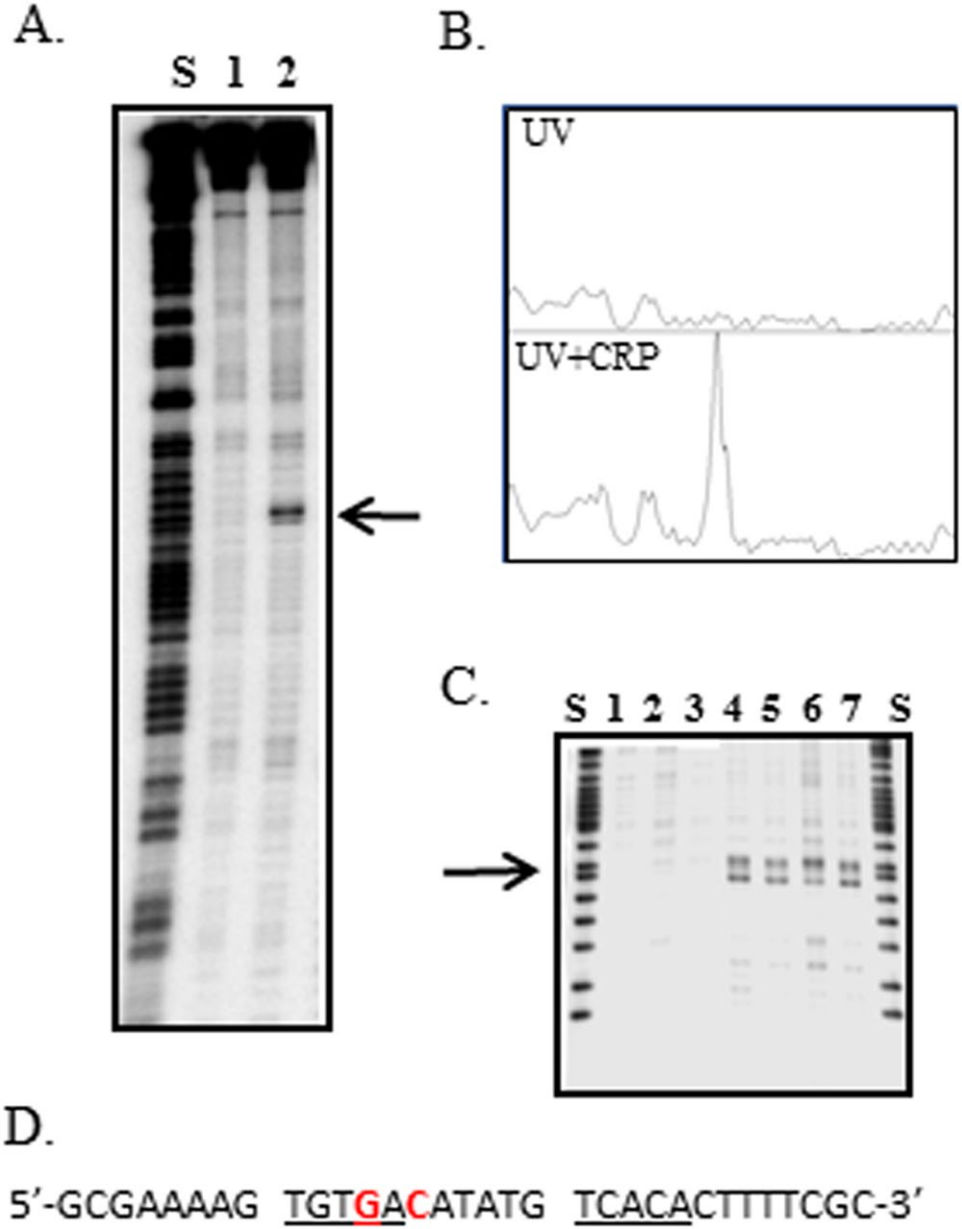
Effect of UV exposure, and presence of CRP-cAMP complex, and additives on integrity of DNA sequence. Panel A: Lane marked “S”: A/G sequence. Lane 1: ^32^P-labelled DNA sequence exposed to 15 min of UV exposure (as indicated in Fig. 1, panel A), in the absence of CRP, in buffer (10 mM Tris–HCl, pH 8.0, 50 mM KCl, 2.5 mM MgCl_2_, 1 mM EDTA, 55 µg mL^−1^ bovine serum albumin, 1 mM DTT, 0.05% NP-40, 2 µg mL^−1^ calf thymus DNA and 50 µM cAMP). Lane 2; as lane 1, except in the presence of 20 nM CRP. Panel B. Representative densitometric scans of lanes 1 and 2 from panel A. Panel C. Lanes marked “S”: A/G sequence. Lane 1: as panel A, but with ^32^P-labelled DNA sequence incubated for 15 min in the dark with no CRP; lane 2: as lane 1 but with 20 nM CRP; lane 3: as lane 1 but with UV exposure for 15 min; lane 4: as lane 3, but with 20 nM CRP and UV exposure; lane 5: as lane 4 but in the presence of 100 mM NaN_3_; lane 6: as lane 4, but with the omission of DTT from the reaction buffer; lane 7: as lane 4, but in the presence of 100 mM mannitol. Panel D. Sequence (underlined) of the CRP binding site on DNA, with the observed sites of cleavage indicated in red.

Experiments were carried out to test whether UV exposure affects DNA integrity (as assessed by denaturing gel electrophoresis) when cAMP-CRP is bound at the strong CRP binding site on the 268 bp DNA fragment. UV exposure of the DNA/CRP/cAMP complex resulted in specific strand cleavage (Fig. 2A, lane 2 and Fig. 2B), which was not observed when DNA alone was exposed to UV light (Fig. 2A, lanes 1 and Fig. 2B). Incubation of DNA/CRP/cAMP complexes in the dark did not induce DNA damage (Fig. 2C, lane 2). This CRP- and UV-light dependent cleavage occurs at specific G/C base pairs (Fig. 2D) in one of the half-sites, in the position where a strong kink is generated in the DNA structure on binding of CRP/cAMP^17^. Omission of cAMP, or denaturing of either the CRP or DNA before irradiation prevented this DNA damage (data not shown). Inclusion of mannitol and NaN_3_ had no effect on the DNA cleavage (Fig. 2, lanes 5 and 7), consistent with a localized and specific oxidation. In the absence of DTT, (usually present in the CRP binding buffer), both cleavage at the specific G/C pair and limited cleavage at other sites was detected (Fig. 2, lane 6). These data indicate that DNA cleavage occurs via two mechanisms: a highly-specific process in the presence of DTT that involves a cAMP-CRP-derived intermediate that reacts at specific nucleotides, and a less-selective and more limited process in the absence of DTT, that involves diffusible oxidants that can react at multiple sites. Thus, the presence of thiols, as would occur *in vivo*, limits DNA damage to a highly-specific, UV-driven, process involving cAMP-CRP-derived species.

### Characterization of UV-induced modification on CRP

Oxidative damage to CRP was examined using two complementary techniques: total amino acid analysis (UPLC with fluorescence detection), and peptide mass mapping using LC-MS/MS, with the former providing data on the overall extents of modification, and the latter data on the sites of oxidation and the identity of the species formed.

**Figure 3:**
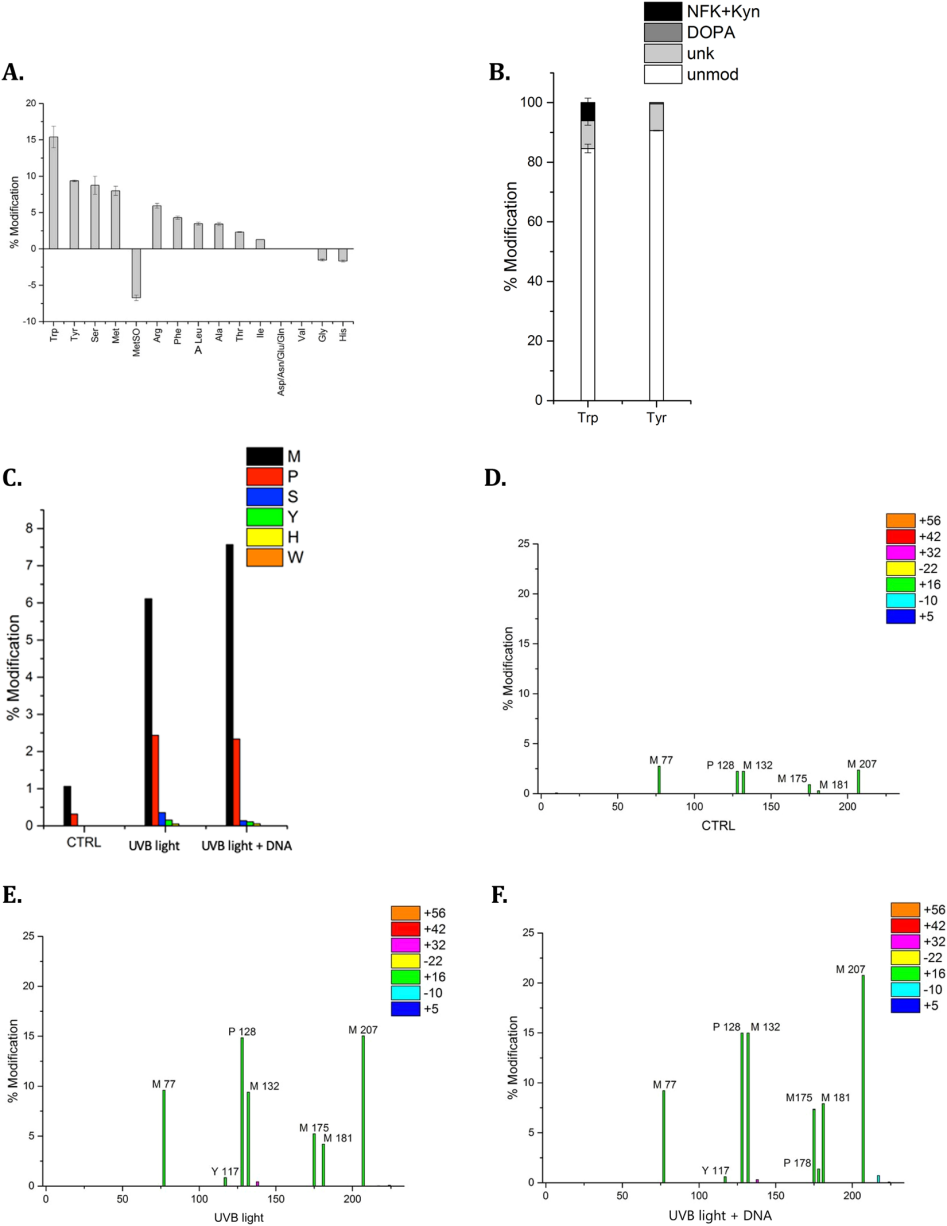
UV exposure alters the chemical composition of CRP in the absence and presence of DNA. Isolated CRP-cAMP complex (0.2 and 50 μM, respectively) was incubated in the dark, or exposed to UV light (λ_max_ 310 nm, 30 nm band width, 6 W m^−2^, normoxia) for 15 min, before analysis using UPLC with pre-column derivatization and fluorescence detection (panels A,B) or LC-MS/MS (panels C–F). Panel A: Changes in amino acid composition determined by acid-hydrolysis and total amino acid analysis. Data are expressed as % modification of the indicated amino acids (positive values indicating loss, negative values indicating formation) relative to the UV exposure control. Mean ± SD from 3 independent experiments. Panel B: Material balance for Trp and Tyr residues determined by UPLC analysis with direct fluorescence detection on acid-hydrolysed UV-exposed CRP-cAMP complex. Levels of unmodified parent amino acid, formation of DOPA, the total of NFK and Kyn (as NFK is converted to Kyn during acid hydrolysis) and unknown products (difference to control values) are indicated. Mean ± SD from 3 independent experiments. Panel C: Extent of modification at different amino acids (Met, M; Pro, P; Ser, S; Tyr, Y; His, H; Trp, W) detected by LC-MS/MS for control CRP-cAMP, UV-exposed CRP-cAMP, and preformed CRP-cAMP-DNA complex exposed to UV. Modifications detected at individual sites (panels D–F) were summed and are expressed as a percentage of the total (native and modified) concentration of the amino acid detected. Panels D–F: Percentage extents of modification at individual amino acids in the CRP sequence (indicated on horizontal axis as position number in sequence). Panel D: control CRP-cAMP complex. Panel E: UV exposed CRP-cAMP. Panel F: CRP-cAMP exposed to UV in presence of DNA (as above). Modifications at the indicated residues are given as the change in *m/z* (−22, −10, +5, +16, +32, +42, +56) detected by MS for the modifications included in the data searches. The majority of the modifications correspond to *m/z* + 16 species assigned to the addition of one oxygen atom (formation of an alcohol at Pro and Tyr; generation of the sulfoxide from Met).

UPLC analysis of native and oxidized CRP showed that UV exposure of CRP resulted in significant modification (relative to controls) to Trp, Tyr, Ser, Met and Arg residues (Fig. 3A). Lower extents of modification (<5%) were detected at other residues. Lys, Pro or Cys could not be quantified using this method. Data for His are not reported, as the 6-His tag on the expressed CRP confounds analysis. A significant increase in methionine sulfoxide, a major Met oxidation product, was also detected. Trp- and Tyr-derived products were identified and quantified using UPLC with direct fluorescence detection, with *N*-formylkynurenine (NFK)/kynurenine (Kyn) detected from Trp (~6%; quantified as the sum due to acid catalyzed conversion of NFK to Kyn), and 3,4-dihydroxyphenylalanine (DOPA; ~0.4%) detected from Tyr (Fig. 3B). The discrepancy between the parent Trp and Tyr lost, and products detected (Fig. 3B) indicates the formation of additional materials.

LC-MS sequence coverage of control and UV-exposed protein was high (~77%; Supplementary Fig. 2), with most of the missing sequence being a single peptide near the N-terminus. Q-TOF-MS analyses of the UV-exposed samples, showed that Met was the most modified (6.1%), followed by Pro (2.4%), Ser (0.4%) and Tyr (0.2%) (Fig. 3C). Analysis using an Orbitrap-Fusion-MS, showed a similar order but slightly higher levels of modification (Met, 16.5%; Pro, 5.6%; Tyr, 1.8%; His, 0.56%). Controls showed only low levels of modification (Fig. 3C). No Trp modifications were detected as these residues occur in non-detected peptides (Supplementary Fig. 2). The decreased extent of modification detected by MS relative to UPLC likely reflects the incomplete sequence coverage, and search strategies that use predefined mass changes (*m/z* +4, +16, +32 Da); uncharacterized modifications are therefore not detected. Q-TOF MS analysis of cAMP-CRP-DNA complexes exposed to UV light showed a similar pattern of damage, but higher extents of Met modification (7.6%; Fig. 3C). The modifications detected at Met and Pro were *m/z* +16 Da species, consistent with Met sulfoxide and hydroxylation of Pro.

**Figure 4:**
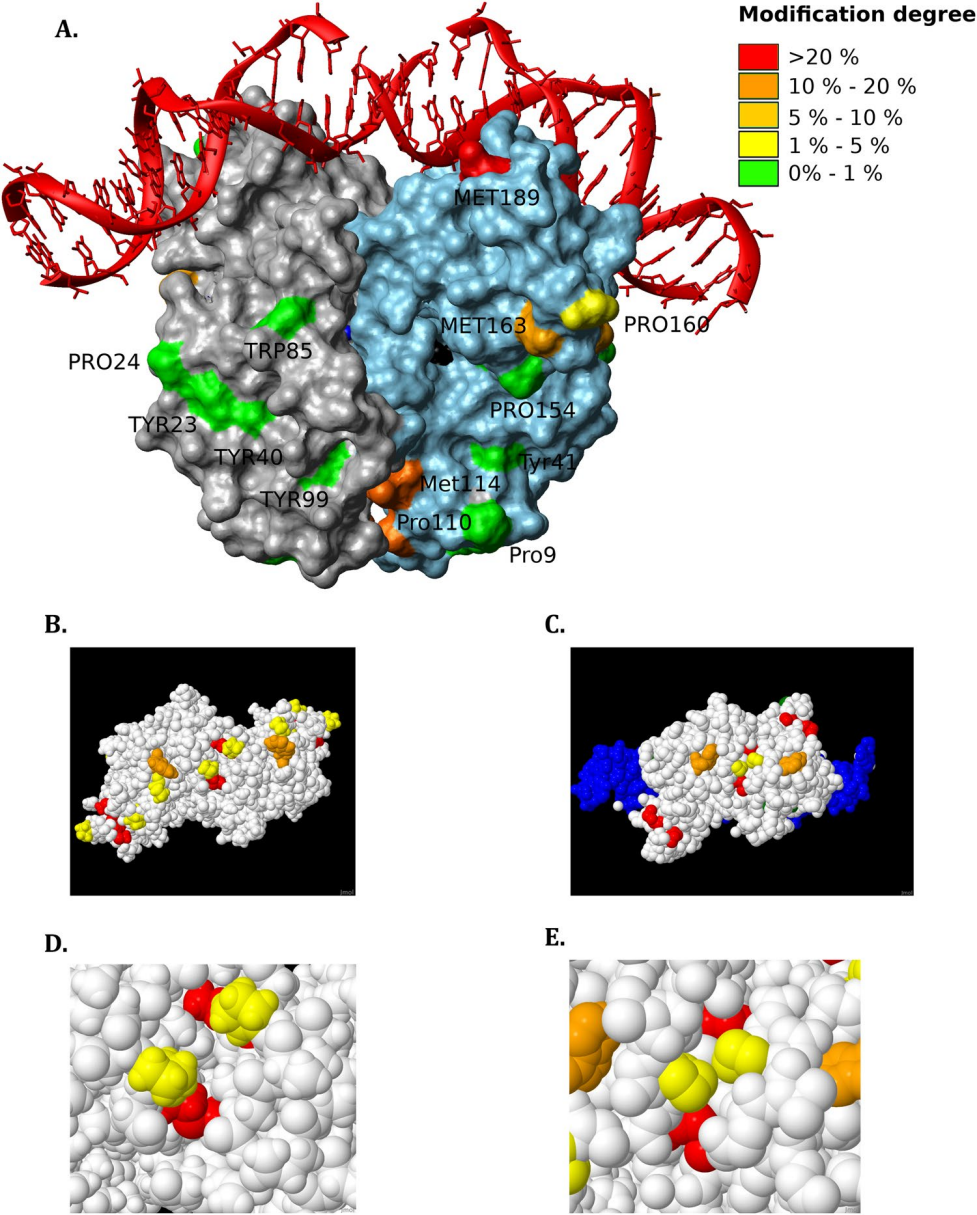
Mapping of UV-induced modifications detected by LC-MS/MS on to the dimeric crystal structures of the CRP-cAMP complex (PDB structure: 2wc2) and the CRP-cAMP-DNA complex (PDB structure: 1O3T). Panel A: Rendering of the sites and relative extents of modifications induced by UV-exposure in the presence of DNA. The two monomeric protein structures are indicated in grey and blue, with the DNA structure indicated in red. The extent of modification at individual amino acids is as indicated, with oxidation hotspots at Pro110/Met114, Pro160, Met163 and in the presence of DNA, Met189. Panels B and C: Rendering of the dimeric structures of the CRP-cAMP complex (panel B, PDB structure: 2wc2) and the CRP-cAMP-DNA complex (panel C, PDB structure: 1O3T). In panel C, the DNA strands are indicated in blue. The positions of the Trp13 (orange), Pro (yellow) and Met (red) residues in the two monomer structures is indicated. Comparison of panels B with C indicates the conformational changes in the CRP structure that occur on DNA binding. Panels D and E: Expansions of the dimer-interface region from panels B and C, indicating the Pro110 (yellow)-Met114 (red) pairs on the individual monomers and the increased proximity of these species at the dimer interface in the DNA-bound structure (panel E) compared to the non-bound (panel D). Modification at these residues, with conversion of Met114 to the sulfoxide and Pro110 to an alcohol are proposed to result in significant steric and electronic interactions that limit binding of UV-oxidised isolated cAMP-CRP to DNA (cf. Fig. 1A), and dissociation of cAMP-CRP from DNA when the bound complex is exposed to UV (cf. Fig. 1B). In panel E, the Trp13 residues (orange) are also indicated as these residues move closer to the Pro110-Met114 residues on DNA binding.

Sequence mapping indicates an uneven distribution of modifications (Fig. 3). Met189 was the most heavily modified, followed by Met59 and Met114, then Met157 and Met163. Low levels of modification at Met120 were also detected using the Orbitrap-Fusion machine. The data for CRP exposed to UV in the presence of DNA, showed higher levels of oxidation at Met114, 157, 163 and 189 (Fig. 3E versus 3 F). In the presence of DNA, Met189 is the most extensively modified residue (20.8%), with this situated very close to the DNA strands. In contrast, Met114, which is also modified to a major extent is positioned close to the dimer interface, and near the cAMP binding site (Fig. 4). The extent of modification at other sites (e.g. Met59, Pro110) was not markedly affected by the presence of DNA.

**Figure 5:**
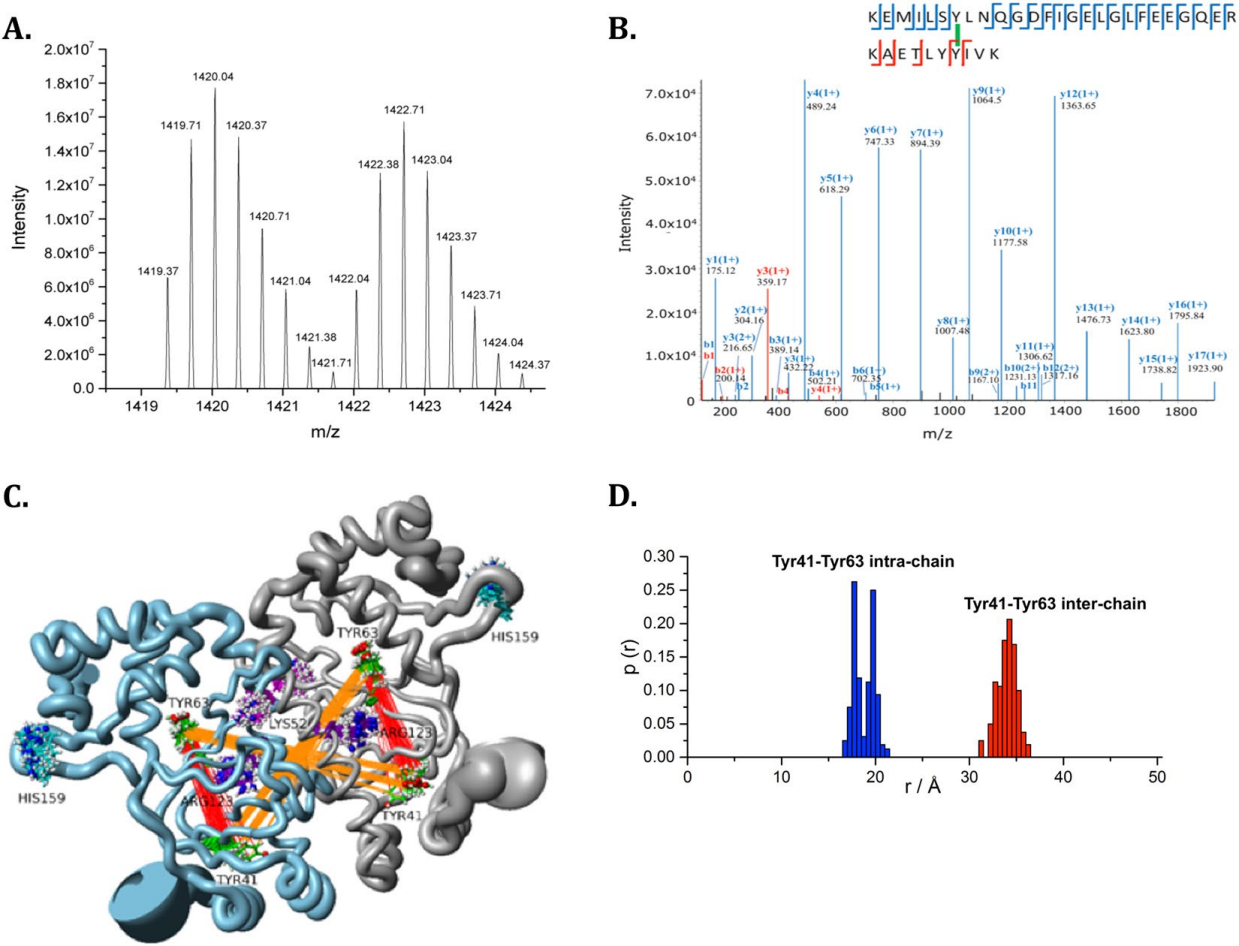
MS detection of inter-molecular di-Tyr cross-link detected in UV illuminated CRP-cAMP complex (see Fig. 4). Panel A: MS spectrum of the triply charged di-Tyr cross-linked peptide KEMILS**Y**LNQGDFIGELGLFEEGQER) (KAETLY**Y**IV), with precursor *m/z* 1419.372 following trypsin digestion in H_2_^16^O, and the corresponding peptides with precursor *m/z* 1426.372 following trypsin digestion in H_2_^18^O. The mass shift between the two envelopes of peaks is diagnostic of the presence of a cross-linked peptide with two new carboxyl termini and the incorporation of four ^18^O atoms. Panel B: MS/MS fragmentation spectrum of the triply charged di-Tyr cross-linked peptide KEMILS**Y**LNQGDFIGELGLFEEGQER) (KAETLY**Y**IV), with precursor *m/z* 1419.372. The fragment ions indicated in blue correspond to *b* and *y* fragment ions from the (longer) α peptide, while the red *b* and *y* fragments correspond to the (shorter) β peptide. The sequence of these two peptides is indicated, together with the site of the di-Tyr linkage between the two Tyr (Y) residues (vertical green line). A number of the detected fragment ions retain this cross-link confirming its location. Panels C and D: Rendering of the structure of the CRP-cAMP complex (PDB structure: 2wc2) indicating the relative positions of the Tyr41 and Tyr63 residues both within the individual monomer chains, and between Tyr41 when these are present on different monomer chains. The corresponding vectors for intra-chain (▪) and inter-chain (▪) cross-links are indicated. In panel D, these vectors are plotted as distance distributions for a putative intra-chain cross-link (blue bars: average 18.7 Å, 16.5 Å shortest) and a putative inter-chain cross-link (red bars: average 34.0 Å, shortest 31.1 Å). These high shortest and median distances (>15 Å) imply that the observed cross-links are inter-molecular in nature and occur between monomers in two different dimers. This conclusion is supported by the surface accessibility of the two Tyr residues in both the DNA-free and DNA-bound forms (Supplementary Fig. 3).

The UV-induced CRP cross-links (Fig. 1D) were characterized using MS with H_2_^18^O labelling (reviewed^18^). This method provided strong evidence for a di-Tyr cross-link between Tyr63 and Tyr41 in a cross-linked peptide (KEMILS**Y**LNQGDFIGELGLFEEGQER) (KAETLY**Y**IVK); this species was not detected in controls. This cross-linked peptide showed the expected −2.01 Da mass difference compared to the sum of the parent peptides, and a theoretical mass close to the experimental (4255.143 vs 4255.092 Da; error 11.9 ppm). The MS spectrum showed the expected +8 Da mass shift (at m/z 1419.37) for a peptide with two carboxyl termini following H_2_^18^O digestion (Fig. 5A). MS/MS analysis (Fig. 5B) revealed multiple fragment ions that retain the cross-link site confirming the identification (b_9_-b_12_ for the longer α peptide; y_4_ for the shorter β peptide). The distance distribution of the atoms forming this cross-link (Fig. 5C,D), are consistent with this being *inter-protein* (i.e. a cross-link between two chains on different dimer molecules), as the average calculated distances between these residues for a putative *intra-chain* link is 18.7 Å (shortest 16.5 Å), for an *inter-chain* link 34.0 Å (shortest 31.1 Å), values that are much larger than those over which such cross-links are likely to form (cf. data for zero-link chemical cross-linking reagents^19^). This conclusion is supported by the surface accessibility of these residues in CRP structures (PDB structures 1o3t, 3hif and 2wc2), and the oligomer formation detected by SDS-PAGE.

## Discussion

UV light, and particularly UVC, is widely used as an environmentally-sensitive sterilization and bactericidal agent in the health sector, and in water and waste stream disinfection. UVB light, (λ 280–315 nm) can initiate direct DNA damage and formation of oxidized nucleobases, cyclobutane pyrimidine dimers, and endonuclease-sensitive sites^20^. Proteins are also major UV absorbing species in biological systems due to both their abundance (5–10 mM in mammalian cells; 1–3 mM in plasma), and significant UVB absorption bands in the 270–290 nm region from Trp, Tyr and cystine residues^12–15^. Some proteins have longer wavelength absorptions arising from interaction of Trp residues with metal cations or charged side-chains^21^, or the presence of co-factors^12^. The high abundance of proteins makes these major *targets* for oxidants^16^, with Trp, Tyr, Cys, cystine, His, and Met side-chains being particularly prone to modification^13,14,16^.

As CRP regulates a large number of transcription units in *E. coli*^1^, and is a global regulator in other gram negative bacteria, UV-mediated damage to the CRP-DNA complex may have significant biological effects. The data presented here indicate that isolated cAMP-CRP is sensitive to biologically-relevant UVB and UVA wavelengths (280–360 nm) and doses, with this generating chemical modifications on specific side-chains, and structural changes (dimer/oligomer formation). Significant UV-induced modification was detected at Met, Trp, Tyr, Pro, Ser and Arg. MS peptide mass mapping indicate that specific Met, Tyr, and Pro residues are modified, with large differences in parent loss and % conversion to products between sites. The extent of modification at individual Trp sites could not be determined, but the overall extent of Trp oxidation was significant, with a proportion of this being NFK and Kyn, together with other uncharacterized materials. Oxidation at Met yielded the sulfoxide, and Tyr oxidation yielded both DOPA and dityrosine. Unusually, significant modification was also detected at Ser and Pro, with these not being typical UV targets. Some of the Ser loss may arise from their proximity to the bound cAMP^22^. Products from Ser (probably carbonyls) were not detected, as these were not included in the MS modification database. The products from Pro were detected as *m/z* +16 species, consistent with the formation of alcohols (hydroxyPro^23,24^).

These chemical modifications are consistent with the observed structural changes. SDS-PAGE data indicates significant levels of non-reducible cross-links, supported by the MS detection of a covalent Tyr63-Tyr41 (dityrosine) link, assigned as an inter-molecular linkage, as the calculated intra-chain and inter-chain distances are too large for such species, unless the protein adopts conformations markedly different to those of free CRP, or DNA-bound CRP. Whether dityrosine is the *sole* type of cross-link is however unclear.

The binding of CRP to DNA is cAMP dependent, with no gel shift detected in the absence of cAMP. However, UV-induced modification of cAMP-CRP had a marked effect on native cAMP-CRP binding to DNA. It is well established^8^ that cAMP-CRP binding to DNA is associated with structural changes at the dimer interface, with the monomer units rotating relative to each other (Fig. 4B,C). Interestingly, two of the most heavily modified residues, Met114 and Pro110, are in close proximity both to each other, and to the respective residues on the other monomer, at this interface (Fig. 4B,C). The distance between the imine nitrogen on Pro, and the sulfur on Met calculated as 8.9 Å. Conversion of the Met residue to the sulfoxide, or addition of an OH group to the Pro ring, is predicted to provide significant adverse steric and electronic interactions between these sites, thereby preventing the rotation and structural changes required for binding of cAMP-CRP to DNA. These residues are close to Trp13, with the distance between C2 of Trp13 and imine nitrogen of Pro110 ~5.7 Å in the non-DNA bound structure. This proximity rationalizes the high extent of modification at these residues. Extensive modification was also detected at these residues when the cAMP-CRP complex was bound to DNA *before* UV exposure. Oxidation at these same Met and Pro residues in the DNA-bound complex, and their resulting unfavourable interactions, may promote dissociation of the dimer from the DNA and rationalize the decreased binding seen in the gel shift assays (cf. Fig. 1A, lanes 4 versus 7).

The modifications detected on both the protein, and also on the bound DNA when the cAMP-CRP-DNA complex was exposed to UV light, occur in a selective and localized manner. Thus, some of the Met, Tyr and Pro residues present in CRP were not modified, or only to a limited extent, whereas others are extensively altered (Fig. 3E,F). A marked selectivity was also detected for DNA chain cleavage, with this only detected at G and C residues within the binding DNA sequence despite the presence of alternative G/C sites. This DNA strand cleavage required both UV light and the protein, pointing to highly-selective protein-mediated events.

The requirement for O_2_, and the lack of effect of mannitol, is consistent with a role for ^1^O_2_ generated via type 2 photochemistry, probably arising from initial light absorption at either cAMP, or more likely the Trp residues. cAMP absorbs light with λ_max_ ~257 nm with a rapid tailing to higher wavelengths and little significant absorption > 280 nm. It should also be noted that crosslinking occurs in the absence of cAMP. In contrast, Trp residues typically have λ_max_ values at ~280 nm with a significant tail up to ~310 nm. However, λ_max_ for Trp residues varies with solvent polarity, pH, metal ion, cations and nearby charged groups; thus some Trp residues show absorption bands in the visible region^21^. These data indicate that the Trp residues in CRP are the likely UV-absorbing chromophores.

Freely-diffusible ^1^O_2_ would be expected to react rapidly with all accessible target residues – both on the protein and the DNA chains, which is not observed. Furthermore, the presence of other materials in the reaction buffers, including DTT, would be expected to remove a large proportion of free-diffusible oxidants. Thus, we propose that the selective damage detected at the dimer interface, and also to the bound DNA chains, arises from the reaction of some of the ^1^O_2_ formed at Trp, with the sulfur atoms of Met114 and Met189 to give persulfoxides [-S^+^(OO^−^)-]. These are analogues of hydroperoxides, are well-established intermediates in sulfur oxidation by ^1^O_2_^25^, and selective oxidants. Reaction of these species with a target results in the formation of Met sulfoxide from the persulfoxide, and oxidation of the target. Such protein-bound, Met-derived persulfoxides formed by ^1^O_2_, would therefore rationalize the oxidation at Met114/Pro110 at the dimer interface, and Met189 and the neighbouring G/C bases in the bound DNA.

Overall, these data indicate that exposure to low levels of UVB and UVA light (λ 280–360 nm) can result in highly selective and specific damage to cAMP-CRP complexes, which then affects the binding of the complex to its target DNA sequence. Furthermore UV-induced oxidation of the DNA bound complex appears to both promote dissociation of the bound CRP, and induce site-specific cleavage of the bound DNA. These data may provide a mechanism for damage to Gram negative bacteria by UVB light via interference with the critical action of CRP in gene transcription in these species.

## Materials and Methods

### Protein purification

The *E. coli crp* clone (pASKA*crp* JW5702) was acquired from the ASKA (GFP-) collection. The version used contained a His-tag and no GFP. *E. coli* CRP was purified from BL21(DE3) containing the pASKA*crp* JW5702 plasmid in which the *crp* coding sequence is cloned under *lac* promoter control in the His-tag vector pCA24N^26^. Cells were grown in LB medium supplemented with 30 μg mL^−1^ chloramphenicol, and *crp* expression was induced at OD_600_ 0.6 with 1 mM isopropyl-β-d-thiogalactopyranoside. Cells from 1 L were then harvested by centrifugation and the pellet frozen overnight at −20 °C. Native CRP was purified by resuspending the pellet in lysis buffer (50 mM sodium phosphate, 300 mM sodium chloride and 10 mM imidazole supplemented with a protease inhibitor cocktail) followed by sonication at 4 °C. Insoluble material was removed by centrifugation (10 000 *g*, 25 min), and the supernatant was then incubated with nickel-nitriloacetic acid agarose beads for 1 h at 4 °C, with gentle rocking. The agarose beads were then loaded in to a column and washed twice with four column volumes of wash buffer (50 mM sodium phosphate, 300 mM sodium chloride and 10 mM imidazole). The CRP was then eluted and collected using elution buffer (50 mM sodium phosphate, 300 mM NaCl and 250 mM imidazole). Purified CRP protein was then dialyzed twice against 5 L dialysis buffer (50 mM sodium phosphate, 200 mM KCl, 5 mM β-mercaptoethanol, pH 8.0) and stored at −80 °C. CRP purity was assessed using SDS–PAGE with Coomassie staining, and the protein concentration was determined by the BCA method. CRP protein lacking the poly-histidine tag was prepared as described previously^27^.

### SDS gel electrophoresis

Protein samples for SDS gels were added reducing loading buffer containing 1 mM DTT 10% SDS, 50% glycerol and 0.1 mg mL^−1^ bromophenol blue. The samples were heated for 5 min at 90 °C and loaded directly on the gel at 1.5–3 μg per lane. SDS-PAGE was carried out on 1 mm NuPAGE NOVEX 4–12% Bis-Tris-HCl gels using NuPAGE Tris-HCl SDS Running Buffer at 150 V (40–55 mA) for 45 min. Protein bands were visualized by staining with InstantBlue.

### Plasmid and DNA fragments

Construction of the plasmid p309 containing a strong CRP consensus site has been described previously^28^. A 268 bp ^32^P-labeled EcoRI-PvuII fragment from p309 was subject for gel electrophoretic mobility shift assay and for analysis of DNA oxidation on denaturing sequencing gels. ^32^P-labelled DNA fragments were produced using T4 polynucleotide kinase or Large Fragment of DNA Polymerase I (Klenow). The labeled DNA fragments were gel purified and subject to electroelution.

### UV irradiation

The protein complexes were irradiated in the indicated buffers in open containers, with a liquid depth of ~ 3 mm without stirring, for the indicated time using a Philips TL20W/12 light tube (spectral envelope between 280–360 nm, λ_max_ 310 nm, ~30 nm band width, 6 W m^−2^). The emission spectrum of these tubes can be found at: https://www.assets.lighting.philips.com/is/content/PhilipsLighting/fp928010001230-pss-global

### Gel electrophoretic mobility shift assays

For specific binding to DNA, CRP at the indicated concentrations were incubated with ^32^P-labeled DNA fragments in 20 μL of CRP binding buffer (10 mM Tris-HCl, pH 8.0, 50 mM KCl, 2.5 mM MgCl_2_, 1 mM EDTA, 55 μg mL^−1^ bovine serum albumin, 1 mM dithiothreitol, 0.05% NP-40, 2 μg mL^−1^ calf thymus DNA) containing 100 μM cAMP for 15 min at 21 °C. After incubation, 10 μL of loading buffer (CRP binding buffer containing 50% glycerol, and 0.1 mg mL^−1^ bromophenol blue) was added, and samples were immediately loaded on 6% polyacrylamide gels (55:1). Following electrophoresis, the CRP–DNA complexes were detected by autoradiography or exposure to phosphor imager storage screens.

### Analysis of DNA oxidation products on denaturing sequencing gels

The protein-DNA complexes were irradiated as described above. Thereafter the samples were precipitated by 2 volumes of ethanol and centrifuged for 15 min. The dried pellet was dissolved in 6 µL of formamide, 90 mM Tris-borate, and 1 mM EDTA at pH 8.3 containing xylene cyanol and bromophenol blue and the samples were heated at 90 °C before loading 2 µL onto 8–10% denaturing polyacrylamide gels (19:1 acrylamide/methylenebisacrylamide). DNA oxidation products were detected by autoradiography or exposure to phosphor imager storage screens.

### Amino acid analysis by UPLC with fluorescence detection

Samples were prepared and subjected to acid hydrolysis using 4 M methanesulfonic acid as previously^29,30^. The resulting amino acid mixtures were subjected to pre-column derivatization using *o*-phthaldialdehyde and separated by UPLC with eluted materials detected by fluorescence (λ_ex_ 340 nm, λ_em_ 440 nm). Identification and quantification were made versus standards, with the data were normalized to the Ala content.

### Detection and quantification of oxidation products by UPLC

Protein samples were hydrolysed and neutralized as described above, then analyzed as described previously^29,30^. Samples were injected on to a reversed phase column (Phenomenex Kinetex EVO) and separated by gradient elution. Product elution was monitored using 2 fluorescence detector channels parametrized according to the retention times of the products and their fluorescence maxima. Data analysis was carried out with Shimadzu Lab Solutions Browser software. Materials were identified and quantified by comparison with commercial standards. To compensate for any losses during processing, data are expressed relative to parent Tyr.

### Mass spectrometric analysis of oxidation products

The detection and quantification of oxidation products was determined on peptides generated by tryptic digestion as described previously^31–33^. Samples (150 µg protein) were prepared as described above, then subjected to buffer exchange into 100 mM ammonium bicarbonate buffer (ABC buffer) using spin filters (10 kDa cut-off). Reduction and alkylation was carried out using tris(2-carboxyethyl)phosphine (TCEP, 10 mM) and chloroacetamide (40 mM) solution in ABC buffer. Residual materials were removed by centrifugation, and the samples digested overnight at 37 °C, using trypsin with 0.2% deoxycholic acid. Peptides were then collected by centrifugation and the removed by precipitation using formic acid. Samples were then analyzed on either a Bruker Impact II ESI-QTOF (Bruker Daltonics) or an Orbitrap Fusion mass spectrometer (Thermo Fisher). For the former separation was carried out using a Dionex Ultimate 3000 chromatography system (Thermo Fisher) with an Aeris Peptide XB C18 Column (25 cm, 2.6 μm particle size, 2.1 mm internal diameter). Samples were eluted using a solvent gradient system over 80 min, using acetonitrile with 0.1% formic acid and 2.5% dimethyl sulfoxide at a flow rate of 200 μL min^−1^. Sampling rate was 2 Hz for MS 4–16 HZ ms2 (IDAS, top20), and a scan range of 150 to 1500 m/z. For the Fusion system, samples were separated on an EASY nLC 100 chromatograph using a flow rate of 250 nL min^−1^ and gradient of solvents A (0.1% trifluoroacetic acid, TFA) and B (80% acetonitrile and 0.1% TFA). Data acquisition was carried out with a universal method consisting of a full MS Orbitrap scan followed by data-dependent high- energy collisional dissociation MS/MS scans. Data analysis was performed using MaxQuant (version 1.5.3.30)^34,35^ with semi-specific tryptic constraints and a 1% peptide level false discovery rate. Carbamidomethylation of cysteine was used as a fixed modification. Data was filtered in order to extract peptide-spectrum matches corresponding to established oxidative modifications using a list of known oxidative modifications^36^, with changes at Met, His, Tyr and Pro as variable modifications^29,31^. The % modification at a particular site was estimated using label-free quantification ratios relative to the parent, as determined using Maxquant. Peaks with changes of >10% were evaluated in respect to elution times, isotopic distribution and MSMS using Skyline (version 4.1)^37^.

### Mass spectrometric analysis of cross-linked peptides

Analysis of cross-linked peptides by MS was performed as described previously^18,32^. Briefly, after tryptic peptides were digested in H_2_^18^O- or H_2_^16^O, they were subjected to solid-phase extraction on activated StageTip C18 reversed-phase discs, and peptides were then dried down (Speedvac concentrator, 3 mins), re-suspended in 10 μl H_2_^18^O and H_2_^16^O water, respectively, and mixed at a 1:1 ratio immediately prior to analysis. Mass spectrometric analysis was carried out on an Orbitrap Fusion mass spectrometer (Thermo Fisher) as described above. Data acquisition was performed either with a universal method characterized by a full MS Orbitrap scan followed by data-dependent high- energy collisional dissociation (HCD) MS/MS scans, or a data-dependent method where a group of signals with mass shifts of 4, 6 and 8 Da are selected for MS/MS. MassAI software (Univ. of Southern Denmark, April 2017) was used to identify and verify cross-linked peptides. The following settings were used: fixed (carbamidomethylation of Cys) and variable (Met, His, and Tyr oxidation) modifications; maximum 2 missed tryptic cleavages; parent mass tolerance 10 ppm; MS/MS peak tolerance 0.02 m/z. Tyr-Tyr, Lys-Tyr, Lys-His, His-His, and Arg-His were selected as potential cross-links.

### Rendering of protein structures

Protein structures 1o3t and 3hif (CRP with and without DNA) were visualized and distance distributions obtained, using MolMol^38^ using scripts generated in GNU Octave^39^, and the PDB NMR structure 2wc2^40^. For distance distributions, all mesomers/chemically equivalent atoms of the coordinate set capable of forming a cross-link were considered, resulting in a sample size of 64–128. Histograms were generated using a bin size of 0.5 Å.

### Statistics

Data are presented as means ± SD from three replicate independent experiments, with errors propagated when data are normalized to another parameter. Statistical analysis was carried out using the packages available in Excel with p < 0.05 taken as significant.

## Supplementary information

Supplementary Information.

## Data Availability

The data that support the findings of this study are available from the corresponding author upon reasonable request.
