# Supplementary material for: UV oxidation of cyclic AMP receptor protein, a global bacterial gene regulator, decreases DNA binding and cleaves DNA at specific sites
Source: Sci Rep. 2020 Feb 20;10:3106. doi: 10.1038/s41598-020-59855-x (PMC7033146; doi:10.1038/s41598-020-59855-x)
Supplement: Supplementary file 1 — Supplementary Information. [file 41598_2020_59855_MOESM1_ESM.pdf]

UV oxidation of cyclic AMP receptor protein, a global bacterial gene regulator, decreases DNA binding and cleaves DNA at specific sites

Fabian Leinisch [1], Michele Mariotti [1], Sofie Hagel Andersen [2], Søren Lindemose [2], Per Hägglund [1], Niels Erik Møllegaard [2]*, Michael J. Davies [1]*

SUPPLEMENTARY DATA

[1] Dept. of Biomedical Sciences, and [2] Dept. of Cellular and Molecular Medicine, Panum Institute, University of Copenhagen, Copenhagen 2200, Denmark

* Joint senior authors. E-mail: nielsem@sund.ku.dk and davies@sund.ku.dk

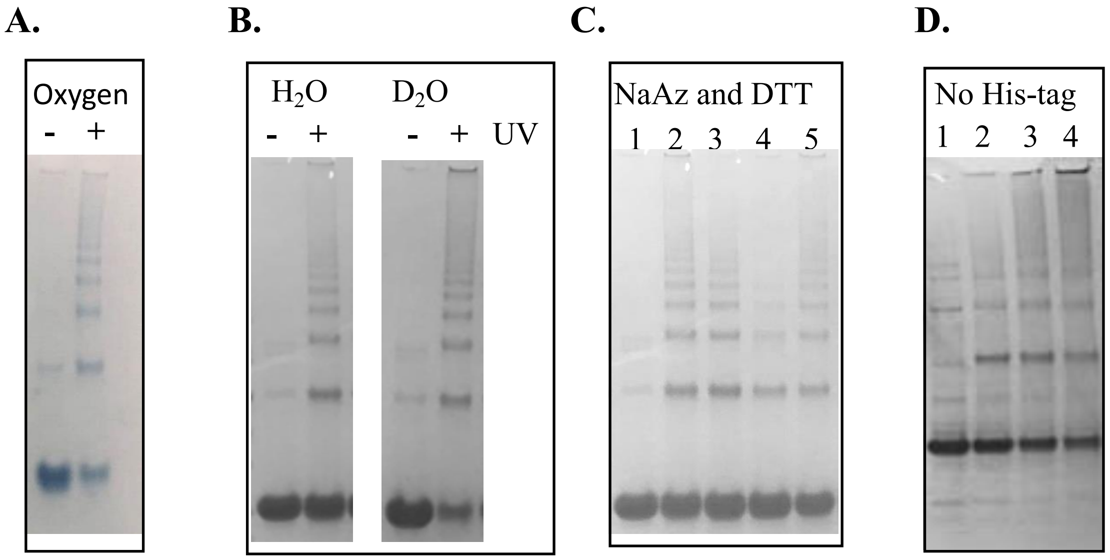

Supplementary Figure 1. Effect of the absence and presence of O_2, the effect of D_2O, NaN_3 and DTT, and the presence of the poly-histidine tag, on the UV-induced oligomerization of isolated CRP-cAMP.

Panel A: Isolated CRP (5 µM)-cAMP (50 µM) complex was exposed to UV irradiation for 20 min (as Figure 1C) under either a N_2 atmosphere (gassing for 20 min) or under normoxic conditions. Samples were then separated on SDS-PAGE gels run under reducing conditions (see Materials and methods), before visualization with InstantBlue staining.

Panel B: Samples of CRP-cAMP complex were made up in either H_2O-containing buffers (see Figure 1C), or buffers containing 80% D_2O, 20% H_2O, and then incubated in the dark for 20 mins, or exposed to UV light for 20 min as indicated. Samples were then separated and visualized as indicated in panel A.

Panel C: Samples were prepared and run under normoxic conditions as described in panel A without or with additives as indicated. Lane 1: CRP in the dark; lane 2: CRP exposed to UV; lane 3: CRP exposed to UV in the presence of 20 mM NaN_3; lane 4: CRP exposed to UV in the presence of 100 mM NaN_3; lane 5: CRP exposed to UV in the presence of 1 mM DTT. Samples were then separated and visualized as indicated in panel A.

Panel D: Isolated CRP (2.5 µM, with the poly-histidine tag removed)-cAMP (50 µM) complex was exposed to UV irradiation for 0 (lane 1), 5 (lane 2), 10 (lane 3) or 20 min (lane 4) under normoxic conditions as described for Figure 1C. Samples were then separated on SDS-PAGE gels run under reducing conditions (see Materials and methods), before visualization with InstantBlue staining.

```
VLGKPQTDPT LEWFLSHCHI HKYPSKSTLI HQGEKAETLY YIVKGSVAVL
IKDEEGKEMI LSYLNQGDFI GELGLFEEGQ ERSAWVRAKT ACEVAEISYK
KFRQLIQVNP DILMRLSAQM ARRLQVTSEK VGNLAFLDVT GRIAQTLLNL
AKQPDAMTHP DGMQIKITRQ EIGQIVGCSR ETVGRILKML EDQNLISAHG
KTIVVYGTRG LCGR
```

Supplementary Figure 2. Sequence of CRP, and MS coverage of sequence for native and modified protein. Sequence is given for mature protein and excludes the His-tag on the recombinant protein. Sequence indicated in green was detected by MS; amino acids indicated in black were not detected; amino acid sequence indicated in red was not able to be analysed in details. Sequence coverage of protein was 90.5% for control, 92.2 % for UV treated, and 90.5% for UV treated CRP-DNA complex

(A) (B)

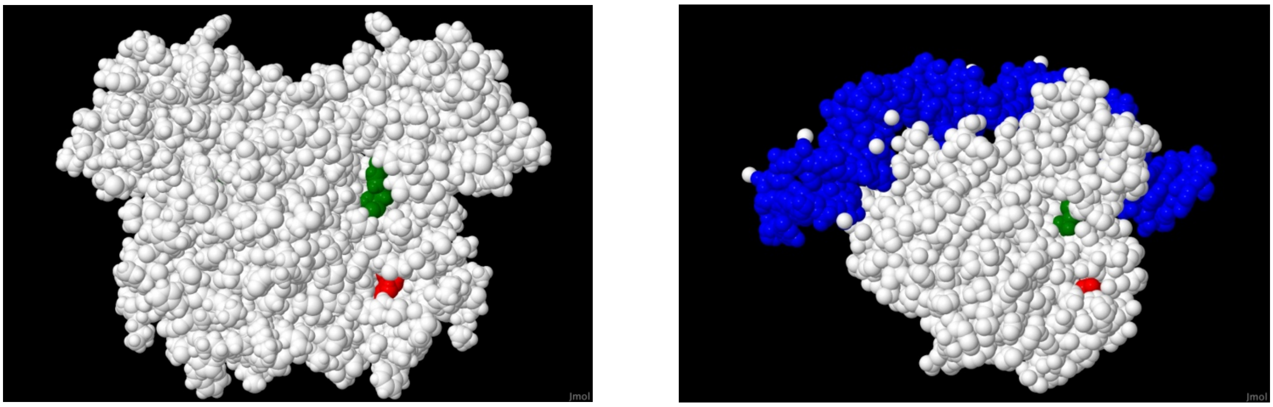

Supplementary Figure 3. Rendering of the dimeric structures of the CRP-cAMP complex (panel A, PDB structure: 2wc2) and the CRP-cAMP-DNA complex (panel B, PDB structure: 1O3T) indicating the relative locations and surface exposure of the Tyr41 (red) and Tyr63 (green) residues.
